# Case report: tuberous sclerosis and persistent hyperplastic primary vitreous

**DOI:** 10.1186/s12886-022-02526-4

**Published:** 2022-07-16

**Authors:** Hayley Wong, Sarah Bowie, Shona Handisides, Julia Escardó-Paton

**Affiliations:** 1 Radiology Department, Middlemore Hospital, Auckland, New Zealand; 2 Ophthalmology Department, Auckland City Hospital and Middlemore Hospital, Auckland, New Zealand

**Keywords:** Tuberous sclerosis, Persistent hyperplastic primary vitreous, Retinal hamartoma, Case report

## Abstract

**Background:**

Persistent hyperplastic primary vitreous (PHPV) in a patient with tuberous sclerosis (TS) has been described in one previous case report in 1999. Otherwise, there is no literature around this potential association. We describe a case of an infant with TS and PHPV.

**Case presentation:**

An 11-month old male was under investigation for leukocoria, microphthalmia and suspected PHPV after being seen in ophthalmology clinic. He presented to hospital with seizures and was diagnosed with TS on imaging. Imaging also showed the known microphthalmia and a mass associated with the lens. Subsequent paediatric ophthalmology review and examination under anaesthesia confirmed microphthalmia, PHPV and a retrolental mass which was thought to represent total retinal detachment or a retinal hamartoma within a retinal detachment.

**Conclusions:**

This is the second case report of PHPV in a patient with TS. The previous case report postulated that the atypical location of the retinal hamartoma was secondary to the abnormal globe development in PHPV.

## Background

Tuberous sclerosis (TS) is a rare neurocutaneous disorder characterised by hamartomas of multiple organs, including the brain, kidney, heart, skin and eyes. It is inherited in an autosomal dominant pattern, though two-thirds of cases are de novo mutations [1]. The reported incidence is between 1 in 5000 to 15 000 live births [2–4]. The most common ophthalmic manifestation of TS are retinal hamartomas, occurring in approximately 50% of patients [5]. Bilateral hamartomas occur in 30% of patients [6]. An unusual ophthalmic finding of a retinal hamartoma in the setting of persistent hyperplastic primary vitreous (PHPV) in a patient with TS has been described in one previous case report in 1999 [7]. This case report describes a patient with similar findings.

## Case presentation

**Fig. 1:**
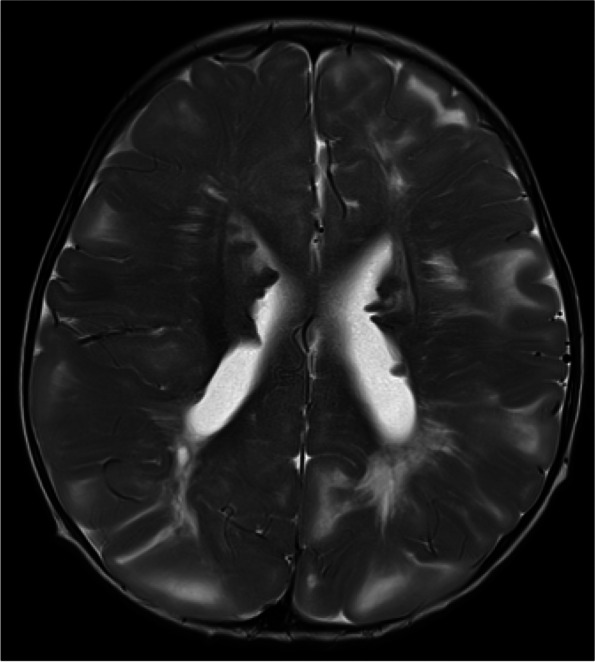
MRI brain axial T2 sequence demonstrates the typical findings of tuberous sclerosis (TS), including subcortical T2 high signal tubers and subependymal nodules lining the lateral ventricles

**Fig. 2:**
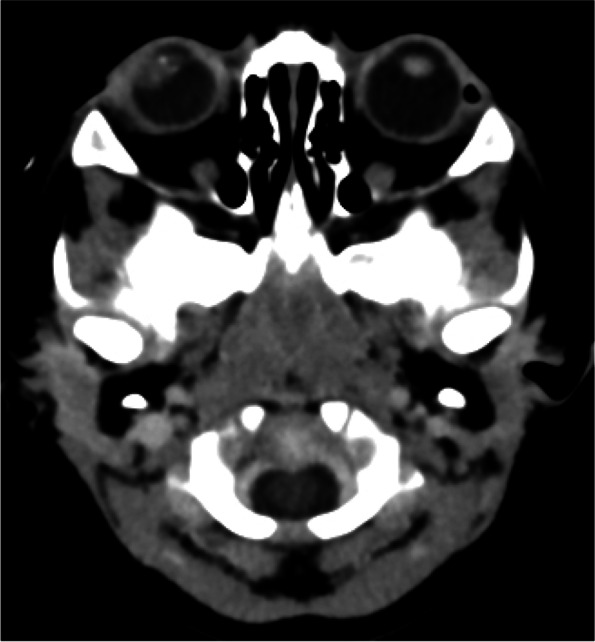
CT head axial non-contrast demonstrates right microphthalmia with a soft tissue mass associated with the lens, with a focus of calcification

**Fig. 3:**
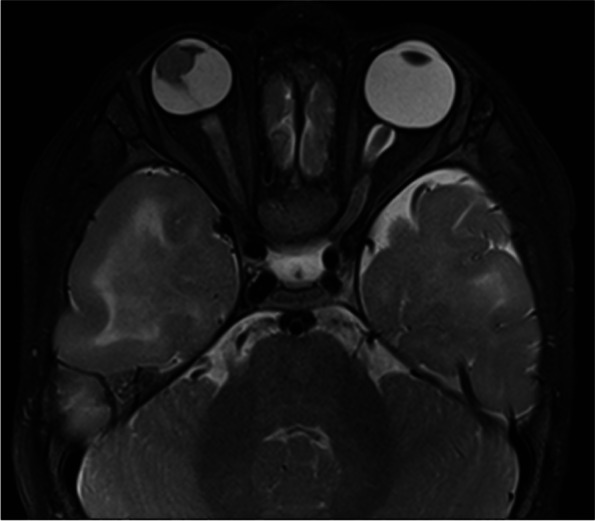
MRI orbits axial T2 and sagittal post-contrast T1 sequences demonstrate right microphthalmia, a T2 intermediate signal mass associated with the lens, extending along a linear structure to the optic disc (Cloquet’s canal). The right lens is small and T2 intermediate signal when compared to the left

**Fig. 4:**
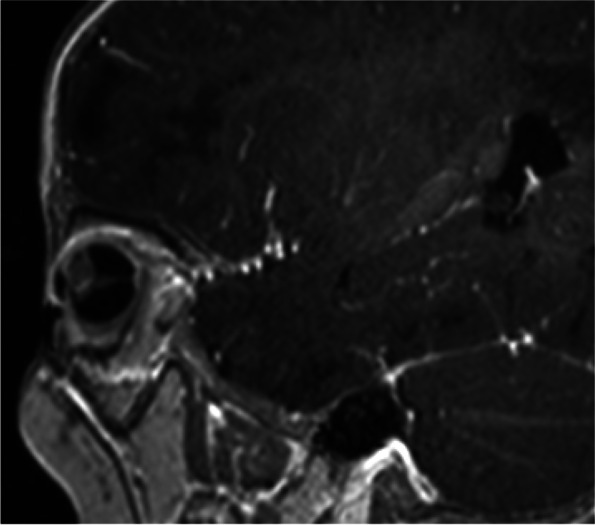
MRI orbits axial T2 and sagittal post-contrast T1 sequences demonstrate right microphthalmia, a T2 intermediate signal mass associated with the lens, extending along a linear structure to the optic disc (Cloquet’s canal). The right lens is small and T2 intermediate signal when compared to the left

**Fig. 5:**
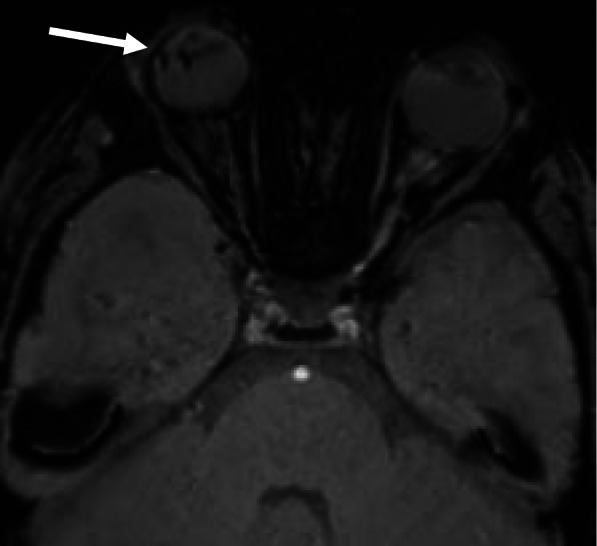
MRI brain axial susceptibility-weighted images demonstrate curvilinear susceptibility artefact (arrow) associated with the right lens, suggestive of retinal detachment

An 11-month old male was referred to ophthalmology for leukocoria and found to have unilateral microphthalmia and suspected PHPV on ultrasound, with a subsequent examination under anaesthesia planned. One month later, he presented to the emergency department with a seizure in the context of a coryzal illness. He was treated with antiepileptics and antibiotics and proceeded to have a CT head and MRI brain which showed findings consistent with tuberous sclerosis; multiple subependymal nodules in the lateral ventricles, some calcified, and diffuse subcortical low attenuation suggestive of tubers (Fig. 1). In addition, there was microphthalmia of the right globe which contained a soft tissue mass lateral to the lens and had foci of calcification on CT (Fig. 2). The mass extended along a linear band of soft tissue (Cloquet’s canal) to the posterior retina in the region of the optic disc. The lens was small, had abnormal intermediate T2 signal and curvilinear septa of susceptibility artefact, suggestive of retinal detachment (Figs. 3, 4 and 5). Following the suspected diagnosis of TS, the patient was found to have hypopigmented lesions on his trunk which were reviewed by the inpatient dermatology team and thought to be consistent with TS. Other investigations included an echocardiogram demonstrating three tumours consistent with TS and an abdominal ultrasound which showed no renal angiomyolipomas. There was no family history of TS or seizures.

Following discharge, outpatient paediatric ophthalmology and examination under anaesthesia confirmed unilateral right globe microphthalmia with cataract and PHPV. The retrolental mass was thought to represent total retinal detachment or a retinal hamartoma within a retinal detachment. The left eye was normal. No treatment was offered due to the poor prognosis of the right eye and the patient continues to have ongoing ophthalmology follow up. The family is awaiting genetics consultation. Informed consent for this report was gained from the patient’s parents.

## Discussion and conclusions

This case report describes a patient with TS with a rare ophthalmic finding of microphthalmia, PHPV and retinal detachment, with the possibility of a retinal hamartoma within a retinal detachment. A similar finding has been described in a case report in 1999 in Canada [7]. A 6-week old was referred for leukocoria and diagnosed with PHPV and TS on examination and imaging. Subsequent enucleation found a retinal hamartoma in an atypical location, explained by PHPV resulting in trapped proliferating elements. The hamartoma was of an unexpectedly large size for the patient’s young age, suggestive of retinal detachment. The contralateral eye had retinal hamartomas typical of TS. There has been no other described association of TS or PHPV since this case report. Our patient’s ophthalmic findings have not been confirmed on histopathology, unlike the previous case report.

PHPV is a rare congenital developmental malformation, caused by the failure of regression of the primary vitreous [8]. It usually occurs sporadically, though there are reports of PHPV in certain rare syndromes, such as trisomy 13, Walker-Warburg syndrome, Norrie disease, osteoporosis-pseudoglioma syndrome [9–12]. PHPV can be complicated by retinal detachment, which is rare in retinal hamartomas [1, 13].

Aside from the aforementioned historical case report, there is no known association between TS and PHPV. Recently, genetic studies have found that microphthalmia-associated transcription factor (MITF) transcription and expression is reduced by mutations in TS genes, TSC1 and TSC 2 [14]. MITF mutations are associated with microphthalmia, retinal degeneration and hyperplasia of the retinal pigment epithelium [15], and we postulate that this may be a possible link.

A strength of this case is that despite the rare occurrence of the conditions, we were able to make these diagnoses under the care of subspecialist paediatric radiologists and ophthalmologists. However, limitations include the absence of histopathological confirmation and genetic analysis currently.

We believe this is the second published case report of a patient with TS and PHPV, resulting in either total retinal detachment or a retinal hamartoma within a retinal detachment.

## Data Availability

The data used/analysed in the current study are available from the corresponding author on reasonable request.

## Abbreviations

PHPV: Persistent hyperplastic primary vitreous
TS: Tuberous sclerosis
CT: Computed tomography
MRI: Magnetic resonance imaging
MITF: Microphthalmia-associated transcription factor

## References

[1] Hodgson N, Kinori M, Goldbaum MH, Robbins SL (2017). Ophthalmic manifestaitons of tuberous sclerosis: a review. Clin Exp Ophthalmol.

[2] Ebrahimi-Fakhari D, Mann LL, Poyro M (2018). Incidence of tuberous sclerosis and age at first diagnosis: new data and emerging trends from a national, prospective surveillance study. Orphanet J Rare Dis.

[3] Osborne JP, Jones AC, Burley MW (2000). Non-penetrance in tuberous sclerosis. Lancet.

[4] Osborne JP, Fryer A, Webb D (1991). Epidemiology of tuberous sclerosis. Ann N Y Acad Sci.

[5] Nelson L, Olitsky S (2013). Harley’s pediatric ophthalmology. The Systemic Hamartomatoses (‘Phakomatoses’) Chapter 22.

[6] Zimmer-Galler IE, Robertson DM (1995). Long term evaluation of retinal lesions in tuberous sclerosis. Am J Ophthalmol.

[7] Milot J, Michaud J, Lemieux N (1999). Persistent hyperplastic primary vitreous with retinal tumor in tuberous sclerosis: report of a case including tumoral immunohistochemistry and cytogenetic analyses. Ophthalmology.

[8] Shastry BS (2009). Persistent hyperplastic primary vitreous: congenital malformation of the eye. Clin Exp Ophthalmol.

[9] Patau K, Smith DW, Therman E (1960). Multiple congenital anomaly syndrome caused by an extra autosome. Lancet.

[10] Dobyns WB, Pagon RA, Armstrong D (1989). Diagnostic criteria for Walker-Warburg syndrome. Am J Ophthalmol.

[11] Prendergast SD, Trese MT, Liu X (1998). Study of the Norrie disease gene in 2 patients with bilateral persistent hyperplastic primary vitreous. Arch Ophthalmol.

[12] Streichen-Gersdorf E, Gassner I, Unsinn K (1997). Persistent hyperplastic primary vitreous in a family with osteoporosis-pseudoglioma syndrome. Clin Dysmorphol.

[13] Céron O, Lou PL, Kroll AJ (2008). The vitreo-retinal manifestations of persistent hyperplastic primary vitreous (PHPV) and their management. Int Ophthalmol Clin.

[14] Cao J, Tyburczy ME, Moss J (2017). Tuberous sclerosis complex inactivation disrupts melanogenesis via mTORC1 activation. J Clin Invest.

[15] Freund C, Horsford J, McInnes RR (1996). Transcription factor genes and the developing eye: a genetic perspective. Hum Mol Genet.

